# The Impact of the Use of e-Partogram on Maternal and Perinatal Outcomes: A Scoping Review

**DOI:** 10.7759/cureus.62295

**Published:** 2024-06-13

**Authors:** Preeti Singh, Anuja Bhalerao

**Affiliations:** 1 Department of Obstetrics and Gynaecology, NKP Salve Institute of Medical Sciences and Research Centre, Nagpur, IND

**Keywords:** digital health, perinatal outcome, maternal mortality, maternal morbidity, obstetric care, healthcare, e-partograph, paper partograph

## Abstract

To overcome shortcomings of the paper partograph, enhance care during labor and delivery, improve record keeping, and help decision-making, several countries have focused on adopting low-cost digital applications. This scoping review highlights the usability and current status of the digital partogram in obstetric care. We conducted a thorough search involving the databases ScienceDirect, PubMed, and Google Scholar for relevant studies from inception till September 2023 by using the keywords “partograph”, “electronic”, and “obstetric” as well as the Boolean operators “AND” and “OR”. Based on the selection criteria, 25 studies exploring the application of electronic partographs (e-partographs) in obstetric care were included in the review.

The majority of the studies examined the efficiency and reported the effectiveness of e-partographs in comparison to paper partographs. The e-partograph has also demonstrated a clear benefit in that the healthcare providers filled out the data, and a reminder mechanism was placed, which might help determine whether the labor process was normal or needed more care. Moreover, an e-partograph was simple to adopt and use for obstetric caregivers and had the potential to save time. To sum up, digital partograph produces superior results to paper partograph. The use of an e-partograph can keep deliveries on track while lowering the need for cesarean sections and prolonged labor. The e-partograph provides essential benefits to its users and also provides a warning system with audible and visual cues that might be utilized to detect difficulties during delivery.

## Introduction and background

It is necessary to periodically and promptly monitor maternal and fetal parameters throughout labor in order to evaluate the health of the mother and the fetus, promote normal labor, identify any problems, and make early clinical decisions. The World Health Organization (WHO) has recommended that skilled birth attendants (SBAs) use the partograph during labor as a tool to document intrapartum maternal and fetal measures appropriately and adequately, provide appropriate labor management education, and diagnose anomalies, for the past 40 years [1].

Medical professionals usually employ the paper partograph, which is the most popular and WHO-recommended labor monitoring tool and aids in the identification of obstetric and fetal problems by providing a visual depiction of metrics for mother and fetal well-being and labor progression [2]. However, traditional paper-based partographs are time-consuming and need skilled healthcare workers for data entry, making them less feasible for usage, especially in developing countries with resource constraints [3]. To overcome these limitations, many countries have turned their attention to digital apps that are low-cost, addressing the shortcomings of the paper partograph, improving treatment during labor and delivery, facilitating decision-making, and streamlining record keeping. Additionally, these digital apps help accelerate decision-making, and reduce the quantity of documentation [4-6]. The increasing use of mobile technologies offers low-resource countries a chance to increase patient access and thereby enhance healthcare services. However, there is scarce data as to how these novel technologies influence outcomes in the field of healthcare. Hence, in the current review, we aim to examine their application and effectiveness.

The WHO states that receiving care from a trained birth attendant before, during, and after childbirth is one of the most important requirements for the prevention of maternal and perinatal deaths [7]. To expedite the referral of a patient to a higher level of care when complications are anticipated or arise during labor, SBA care must be available at all levels of the healthcare system. Partograph is therefore used in addition to this intervention [4]. The partograph is a pictorial record of the fetus's and the mother's progress during labor and it incorporates alert and action lines to prompt the beginning of additional interventions by SBAs monitoring the progress of labor [8].

Partograph is a useful tool for tracking the progress of labor. Specifically, in developing nations, it prevents obstructed labor, which is a major factor responsible for the deaths of mothers and newborns [5,6,9]. It is estimated that 5% of pregnancies globally result in obstructed labor, accounting for 8% of maternal deaths [3,10,11]. The WHO advises that the partograph be used universally for routine labor monitoring during labor since it helps medical professionals diagnose and treat obstructed and prolonged labor more effectively by helping them recognize slow labor progress [12,13]. Furthermore, partograph serves as an "early warning system," assisting in early transfer decisions, hospital intervention decisions, and ongoing monitoring of the effect of treatments to reduce maternal mortality caused by prolonged labor. In short, evaluating the partograph's effectiveness is crucial and it has become a necessary component of standard labor care in most parts of the world.

In the healthcare system, paper-based partographs are prone to various issues such as inaccuracy, delays, and client information loss [3]. Even after years of training and investments in partographs, paper partographs are still underutilized in settings with limited resources [11,14]. The correct use of a partograph to record important maternal and fetal data is essential for achieving favorable pregnancy outcomes. Recently, a digital partograph or electronic partograph (e-partograph) was developed and is being used in several countries to simplify the process of retrieving and storing client and patient data. Moreover, it aids in timely decision-making and decreases the bulk of paperwork [15]. The widespread adoption of mobile technologies has enabled resource-constrained nations to enhance both the quality and accessibility of their healthcare services. However, there is a dearth of information on how it affects labor outcomes. Hence, we conducted this scoping review to examine the application and efficacy of digital partographs or e-partographs.

## Review

**Figure 1:**
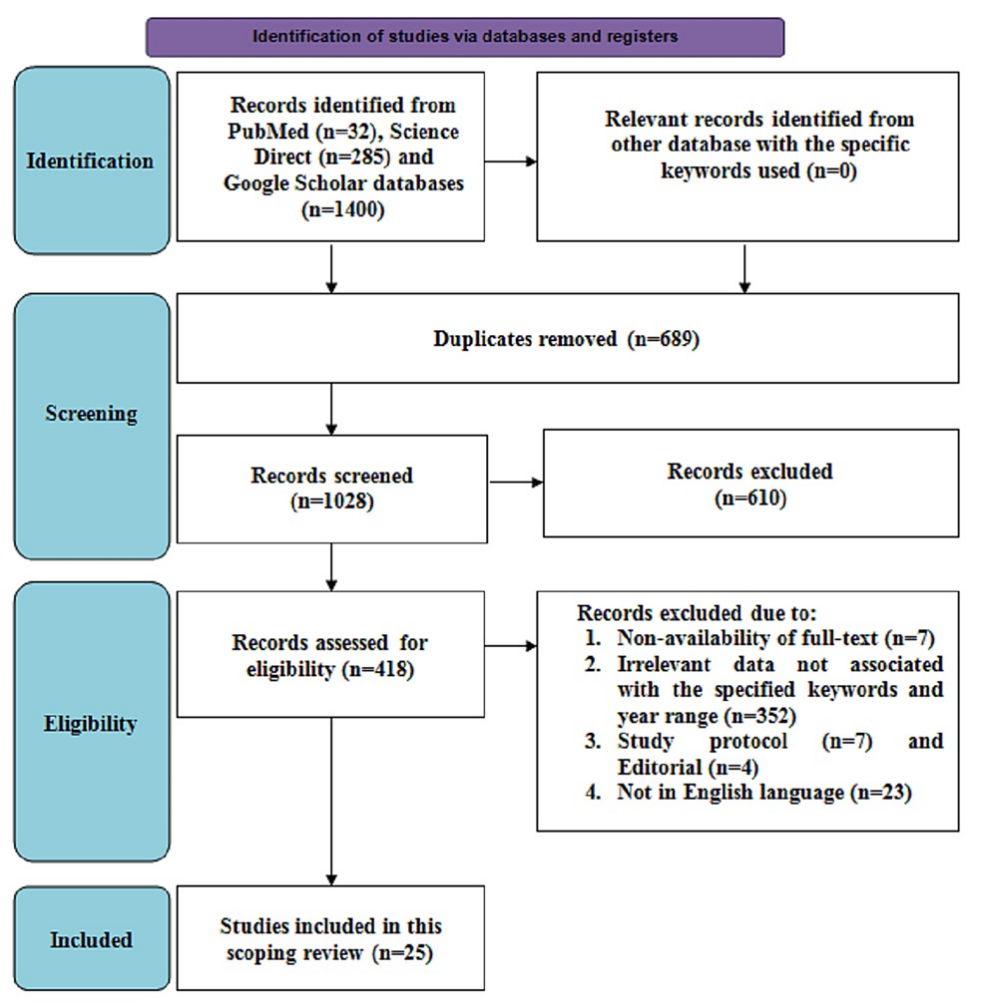
Search strategy for the inclusion of studies

This scoping review adhered to the Preferred Reporting Items for Systematic Review and Meta-Analysis for Scoping Review (PRISMA-ScR) guidelines. ScienceDirect, PubMed, and Google Scholar databases were explored for relevant articles from inception till September 2023. The search involved the keywords “partograph”, “electronic”, and “obstetric” with Boolean operators “AND” and “OR” used in between the keywords. The inclusion criteria were as follows: articles involving brief descriptions of the impact of e-partogram use on maternal and perinatal outcomes, articles with full-text availability, and those published in the English language. A detailed description of the search strategy is presented in Figure 1.

We included a total of 25 studies describing labor monitoring through application-based partographs. A summary of these studies is presented in Table 1.

**Table 1:** Technical aspects and intervention content CHW: community healthcare worker; HIS: health information system; NS: not stated; WHO: World Health Organization

Sr. no.	Authors	Technology/application	Platform	Interoperability/HIS context	Intervention content
1.	Litwin et al. [6], Sanghvi et al. [9], Jain et al. [10]	e-Partogram	Android tablet app	Data accessible at referral facilities	A mobile partograph including auditory alerts, automatic data graphing, and assistance in clinical decisions
2.	Rahman et al. [3], Tadesse et al. [11], Jain et al. [10], Rinkoo et al. [14], Singh and Narwal [4], Tayade and Jadhao [16]	E-partograph	Android smartphone or tablet app	Partograph data monitored remotely	A mobile partograph that produces abnormality alerts and graphs labor progression automatically
3.	Schweers et al. [15]	mLabour	Mobile app	NS	Labor management tool and mobile partograph for registration of patients, measurements, and delivery of records
4.	Singh et al. [17]	prasavGraph	Android smartphone or tablet app	Birth registration with municipal authorities	A mobile partograph that sends abnormality alerts, and keeps digital records
5.	Usmanova et al. [12], Cansdale et al. [18]	ASMAN	Tablet-based	Integrated into government databases	Platform with dashboards, E-learning content, remote support center, and digital case sheets
6.	Andreatta et al. [19]	PPH monitoring	SMS-based	NS	Protocol consisting of numeric SMS for reporting birth outcomes and maternal demographics
7.	Somannavar et al. [20]	Mobile Delivery Timer	Android smartphone app	NS	An application that records audio to mark a baby's birth, crowning, bag-mask ventilation, or crying
8.	Kalibbala et al. [21], Dolk et al. [22]	Birth defect surveillance	Android tablet app	NS	Application for documenting birth outcomes and defects
9.	Ngabo et al. [23], Musabyimana et al. [24]	Rapid SMS	Open-source app	Automatic notification to ambulance service	SMS protocols facilitate communication between healthcare systems and CHWs and are used to record results associated with pregnancy, child, and maternal outcomes
10.	Little et al. [25], Medhanyie et al. [26]	Data collection tool	Smartphone app	Local health authority	An application consisting of an analytics dashboard and maternal healthcare forms
11.	Stroux et al. [27], Martinez et al. [28]	Perinatal monitoring system	Android smartphone app	Open MRS	SMS protocols facilitate communication between healthcare systems and CHWs and are used to record results associated with pregnancy, child, and maternal outcomes
12.	Zaidi et al. [29]	Hayat	Smartphone app	Local health authority	A dashboard to visualize health reports and a mobile application to digitize the entry of data records
13.	Indriani et al. [30]	Maternal referral mobile system	Smartphone app	Data available at a referral center	An application that allows referral hospitals and primary healthcare to communicate about maternal referrals
14.	Sharma et al. [31]	Arc partograph	Web application	Integrated into institutional databases	Based on WHO recommendations, it is a modern, comprehensive, real-time labor monitoring system that incorporates all the stages, including prepartum, partogram monitoring, and postpartum. It also includes maternal health guidelines relevant to each country, for example, LaQshya and DAKSHATA for India

Advantages of e-partograph

The partograph is a tool used to monitor and document labor progress. The key parameters in the partograph are the progression of labor (cervical dilating, contractions, and descent), maternal health (blood pressure, pulse, temperature, urine protein, and acetone), and fetal condition (fetal heart rate, amniotic fluid, and molding). The person filling out the paper partograph is ultimately responsible for its interpretation and completion. Utilizing the e-partograph has several benefits. Additionally, the e-partograph was developed using the inputs provided by each developer and was designed as a tablet-/smartphone-based app connected to a web-based computer device. One of the advantages of employing an e-partograph is that it enables timely decision-making as to whether to intervene or if this delivery can be aided during labor monitoring [24,25].

With the addition of notification capabilities such as audio and visual alerts that display the complications and charging time affecting newborns and mothers, the e-partograph demonstrates various advantages compared to paper partographs in some system applications. Users may make better decisions and take more proactive actions with the assistance of this notification system. Other benefits in some applications include the ability to save filled-in data and create graphs that appear based on that data with different display outcomes. It can be utilized for guidance and patient information sharing at all healthcare system levels. It provides the antepartum, intrapartum, and postpartum period monitoring, in contrast with paper partographs. It reduces the amount of post-delivery retrospective data entry, illustrating the effectiveness of labor monitoring. This demonstrates that there are additional advantages to the e-partograph over the paper partograph. Several inventors have focused on low-cost digital solutions to overcome issues with the paper partograph, enhance documentation, streamline decision-making, and improve care quality, throughout the labor and delivery process [20].

Acceptance of e-partograph

The e-partograph user rate was found to be higher than the paper partograph in both phases (phase 1: 3.31, and phase 2: 15.20) of a prior study. The results were statistically significant after adjusting for parity, mother's education, husband's education, maternal age, religion, gestational age, and fetal sex [3]. Furthermore, by the fifth shift (out of 84 shifts), 93% of SBAs in the study showed a high level of comfort and confidence in using the e-partogram. From the initial client use onward, SBAs reported positive results about the e-Partogram, finding it effective and simple to use. Moreover, SBAs observed that the visual (indicating abnormal measurements) and the auditory (signaling that the measurements were due) alerts were useful and they gained confidence in their ability to interpret and respond to the notifications and reminders [6].

Efficacy of e-partograph 

The use of the e-partogram was associated with a 56% lower likelihood of suboptimal fetal outcomes than the paper partograph and a significantly lower risk of suboptimal maternal outcomes [9]. A previous study reported that the facility-based cesarean section rates showed a declining rate in two hospitals from 43% to 37% and from 36% to 25% respectively, and there was a significant decrease in prolonged labor with the adoption of an e-partograph [3]. Additionally, during phase 1, the prolonged labor rate with the paper version in the two hospitals was 42% and 30% respectively, which decreased to 29% and 7% respectively with the use of e-partograph [3].

Strengths and limitations

The e-partogram enables enhanced decision-making and simple and user-friendly data entry. However, the limitations encountered comprised issues in high-volume labor wards related to data entry. Hence, as a part of the development process, a feedback session with SBAs was performed [6.9]. Similarly, most of them preferred the utilization of e-partographs; however, obstetric care providers were not willing to use them [3,11,10]. Additionally, mLabour users preferred the tablet format in comparison to paper and requested additional features such as tracking of medications. However, some features of the application were customized and were not available for wider use [15]. Similarly, prasavGraph was designed for utilization in places where network connection was poor [17]. Moreover, ASMAN technology was easy to use and showed enhanced job performance, but it demonstrated internet and technology issues, and high caseloads leading to incomplete fields. It involved e-learning written in Hindi and English with visual and audio formats that are readable [12,18]. PPH monitoring training was given in English and the native language but power outages, poor network, and lack of remuneration were encountered [19]. Additionally, most users found that the mobile delivery timer application is easy to use and was beneficial as the Android operating system that was chosen functions on low-cost hardware [20].

The birth defect system does not capture babies born outside the urban facilities [21,22]. Workers who used the rapid SMS feature reported being more proactive in detecting pregnant women and requested financial compensation. However, the limitations faced consisted of high initial costs and the lack of equipment that prevented all the data from being collected [23,24]. For the data collection tool, the forms were found to be comprehensive, and the electronic forms were helpful in comparison to paper forms but the high cost of covering phone airtime was observed, and solar lamps were provided to charge smartphones [25,26]. The perinatal monitoring system was easy to operate but many users were unfamiliar with the technology and low-quality ultrasound recordings were noted. For illiterate users, visual and audio instructions were used to adapt the user interface [27,28]. The Hayat application was found to be feasible with improved efficiency; however, CHWs had to travel long distances to sync data with the central server each week [29]. The maternal referral mobile system demonstrated bugs in its application and focus group interviews were conducted before the development [30].

## Conclusions

According to most of the studies included in our review, the goal of developing an application-based partograph was to streamline the labor monitoring procedure. The delivery outcomes were more effective with the use of the e-partograph compared to the paper partograph. The e-partograph reduced prolonged labor and cesarean procedures, enabling normal delivery. It offers prenatal, intrapartum, and postpartum monitoring. It also enables the documentation of newborn-related factors such as sex, birth weight, Apgar score, and the reason for neonatal ICU admission if necessary, in addition to maternal parameters. Moreover, the e-partograph features a visual and auditory reminder system that helps identify problems during childbirth. It also offers the option of classifying cesarean sections according to Robson's criteria. Additionally, all information was retained and was retrievable, simplifying the documentation system. The fact that the e-partograph was used more frequently than the paper partograph indicates that it has found acceptance among obstetric care providers.
